# Mobile telephone-delivered Contingency Management (mCM) to reduce heroin use in individuals with opioid use disorder (CM4OUD): A feasibility study protocol

**DOI:** 10.1371/journal.pone.0324516

**Published:** 2025-05-28

**Authors:** Carol-Ann Getty, John Strang, Ewan Carr, Jesse Dallery, Nicola Metrebian

**Affiliations:** 1 National Addiction Centre, Institute of Psychiatry, Psychology and Neuroscience, King’s College London, London, United Kingdom; 2 Biostatistics and Health Informatics, Institute of Psychiatry, Psychology and Neuroscience, King’s College London, London, United Kingdom; 3 Department of Psychology, University of Florida, Gainesville, Florida, United States of America; PLoS ONE, UNITED STATES OF AMERICA

## Abstract

**Background:**

Opioid use disorder (OUD) is a major public health issue and recovery is a long-term and complex process. Opioid Agonist Treatment (OAT) including medications such as methadone and buprenorphine, is the first-line medical intervention for OUD, however clinical responses among sub-populations differ and concurrent heroin use among individuals in OAT is reported. Contingency management (CM) is a behavioural intervention involving the application of positive reinforcement (e.g., monetary incentives) contingent upon evidence of positive behaviour change. CM is based on the theoretical principles of operant conditioning and is among the most efficacious psychosocial intervention in promoting substance use-related behaviours, including abstinence from smoking, alcohol and illicit drugs, medication adherence, vaccination uptake and attendance. Technology can be leveraged to expand the reach and accessibility of these interventions, automating key components of intervention delivery, including objective behaviour monitoring and immediate reward delivery. Currently, there are no fully remote CM interventions specifically targeting heroin use among individuals undergoing treatment for OUD, highlighting a critical need for innovation in addressing this complex aspect of substance use. Developing and delivering a fully digitalised app-based CM intervention for reducing heroin use among individuals in treatment for OUD holds considerable potential. This paper provides a protocol for a feasibility study that aims to determine the acceptability and feasibility of conducting a future randomised controlled trial of the clinical effectiveness of app-based CM to encourage heroin abstinence among clients receiving OAT in UK drug treatment services.

**Methods:**

Forty OAT service users in UK drug treatment services who continue to use heroin will be randomly assigned to either (1) OAT plus a smartphone app providing abstinence incentives or (2) standard OAT alone. Participants in the intervention arm will receive financial incentives contingent on heroin-negative toxicology results. Over a 12-week period, participants will receive thrice-weekly push notifications via the smartphone app when an oral saliva test is due. Participants will receive feedback upon submission and verified heroin-negative tests will result in notification of earnings. The primary outcome of this feasibility trial is the number of eligible service users recruited over the 6-month recruitment period. Other feasibility outcomes include intervention adherence, drug screening completion and follow-up rates. Acceptability will be explored among both clinicians and service users. Progression to a larger confirmatory trial will be evaluated based on the pre-specified progression criteria.

**Discussion:**

Research on CM has grown exponentially over the last decade, with remote technologies being leveraged more than ever to expand the reach and scope of these interventions. This study will evaluate the feasibility of a mCM app to support heroin abstinence among OAT recipients. By integrating CM with mobile technology, this approach could enhance treatment accessibility and effectiveness, potentially improving outcomes for a high-risk population.

## Introduction

Opioid use disorder (OUD) is a major public health issue and recovery is a long-term and complex process, with the average time in treatment for opiate problems around two years longer than for other substances [1]. Deaths globally from opioid overdose continue to rise, exceeding 125,000 and accounting for approximately 25% of all drug-related deaths [2]. Half of adults receiving treatment in the UK for substance use are there for problems with opiates. Opioid Agonist Treatment (OAT) including medications such as methadone and buprenorphine, is the first-line medical intervention for OUD. These treatments are strongly supported by evidence showing their effectiveness in lowering mortality and substance use, enhancing physical and mental health, reducing criminal activity, and mitigating HIV risks and associated behaviours [3].

Despite the well evidenced effectiveness of OAT, clinical responses among sub-populations differ and concurrent heroin use among individuals in OAT is reported. In England, the rate of illicit opiate abstinence after three and six months of OAT was estimated as 46% and 48%, respectively [4]. Several risk factors are associated with continued heroin use during OAT, including suboptimal doses [3], little to no psychological therapies [5] and personal situations and environments including continued connections with drug using peer groups [6,7]. Continued heroin use is associated with high treatment drop-out rates, underscoring the need for comprehensive and innovative interventions to address this complex issue [8].

Contingency management (CM) is a behavioural intervention involving the application of positive reinforcement (e.g., monetary incentives) contingent upon evidence of positive behaviour change. CM is based on the theoretical principles of operant conditioning and is among the most efficacious psychosocial intervention in promoting substance use-related behaviours, including abstinence from smoking, alcohol and illicit drugs [9–15], medication adherence [16,17], vaccination uptake [18] and attendance [19]. CM targeting opioid abstinence in service users undergoing treatment for OUD was associated with a medium-large effect size on abstinence versus a control intervention [17].

Despite CM’s well-established research evidence base and the UK’s National Institute for Health and Care Excellence (NICE) recommendation to evaluate and integrate it into addiction services, the UK has a limited track record of applying these interventions. One potential solution to significantly expand access to CM while minimising the strain on resources and staff is the remote delivery of CM. The challenges of implementing CM in real-world settings, particularly in regions with budget constraints and logistical barriers such as UK drug and alcohol treatment services, have been well-documented [20–23]. Technology offers a way to overcome these challenges by automating key components of the process, such as objective behaviour monitoring and immediate reward delivery, thereby ensuring treatment fidelity while alleviating pressure on overburdened healthcare systems [24].

Previous work undertaken by the research team and others, suggests that digital CM is feasible, acceptable and effective in promoting positive behaviour change, including adherence to prescribed medications [25,26], clinical appointment attendance [25] and abstinence from alcohol [27–33], smoking [33–40] and other substances [25,41]. High levels of acceptability towards remote monitoring of behaviour and delivery of reinforcement among service user recipients [42,43] and non-recipients [44] were also found, with 81% reporting to be in favour of incentive programmes. Importantly, 96% of service users owned a mobile telephone (85% of which were smartphones), suggesting it is a potentially viable approach. Consultations with policy and clinical stakeholders have provided further support for the development and implementation of these interventions within UK drug and alcohol treatment services [45].

However, a significant gap exists in the space of opioid use. Currently, there are no fully remote CM interventions specifically targeting heroin use among individuals undergoing treatment for OUD, highlighting a critical need for innovation in addressing this complex aspect of substance use. Developing and delivering a fully digitalised app-based CM intervention for reducing heroin use among individuals in treatment for OUD holds considerable potential. This novel approach has the capacity to significantly broaden the scope and reach of CM treatment, supporting clients undergoing OAT to reduce and abstain from continued heroin use. This paper provides a protocol for a feasibility study that aims to determine the acceptability and feasibility of conducting a future randomised controlled trial of the clinical effectiveness of app-based CM to encourage heroin abstinence among clients receiving OAT in UK drug treatment services.

## Methods

This study was prospectively registered (ISRCTN60538178) and has been reviewed and given favourable opinion by West Midlands – South Birmingham Research Ethics Committee (24/WM/0216). We report this protocol (V1.2, 8.1.25) using the SPIRIT guidelines for reporting of intervention trials (see Supporting Information). Protocol amendments will be agreed and approved by the sponsor and REC. The project is currently in development phase and due to commence recruitment on 1^st^ July 2025. Last participant will be recruited on 31^st^ December 2025 and data collection completed by 25^th^ March 2026. Results are expected to be available by 1^st^ December 2026.

### Aims and objectives

This study aims to determine the acceptability and feasibility of conducting a future randomised controlled trial of the clinical effectiveness of app-based Contingency Management (mCM) to encourage heroin abstinence among clients receiving OAT in UK drug treatment services.

Feasibility study objectives are as follows:

Primary objective:

1. Determine the number of eligible service users and recruitment rates.

Other objectives are to:

1. Determine adherence to the intervention (e.g., user engagement, responses to prompts, uploads);
2. Determine follow-up rates at 12 weeks;
3. Explore intervention acceptability among participants (including those not receiving the mCM intervention);
4. Explore intervention acceptability among treatment providers;
5. Determine app usability using the System Usability Survey [46];
6. Explore the suitability and participant acceptability of objective measures for the primary clinical outcomes (i.e., heroin abstinence);
7. Characterise aspects of the primary outcome needed for a sample size calculation for a larger trial (including an estimate of the intraclass correlation).

### Design and setting

The feasibility study will use an individually randomised controlled design, where service users will be recruited and randomly assigned (1:1) to one of the conditions below over a 12-week period:

A mCM (app-based CM + OAT): Smartphone App plus incentives for objective verification of heroin abstinence
B TAU: Treatment as Usual (OAT only)

The trial design has been created to reflect the future confirmatory trial in which the novel intervention “Mobile Contingency Management” (mCM) will be compared to a control condition (TAU) in which no CM will be received.

Two sites will be recruited: one from South London and the Maudsley NHS Foundation Trust (SLaM NHS) and one from a non-NHS service. Drug services will be eligible if they provide treatment to individuals with OUD.

To advise on all aspects of study design and implementation, a PPIE group was convened. During both study conception and app design, we consulted with the PPIE group, who highlighted positive feedback on CM for drug abstinence and remote testing benefits like independence. Participants liked the app’s simple design and potential to aid behaviour change. Discussions about intervention specifics, including incentive magnitude, testing window and clinician involvement will be continued. The PPIE group will be given the opportunity to engage with the app and provide feedback once developed. The group will be consulted with at key points during the trial.

### Eligibility

The eligibility criteria for enrolling service user participants will include:

1. Receiving OAT (methadone or buprenorphine, including pro-longed release);
2. Self-reported heroin use at least 1 day/week;
3. Aged ≥ 18 years;
4. Able to operate an Android or iOS smartphone with acceptable capability;
5. Willing to receive 12-week CM intervention;
6. Able to provide informed consent.

Service users will be excluded if currently participating in any other research studies.

### Recruitment

Service users (n = 40) receiving OAT who continue to use heroin will be recruited over 6 months (July to December 2025). Individuals will be identified by their keyworker at participating clinics, provided with the Study Summary Sheet and asked to sign the Permission to Contact form. The researcher will contact those service users and arrange an appointment. During this appointment, service users will be screened for eligibility and, if eligible, provided with the Participant Information Sheet. Reasons for ineligibility for the trial will be recorded. Participants will be enrolled in the study, complete baseline assessments and be randomised to one of the conditions described below. Participants will be reimbursed £10 for their time.

### Intervention

Participants will continue to receive OAT (methadone or buprenorphine, including pro-longed release) as part of their treatment for OUD. Each participant will be randomly allocated (1:1) to receive one of two treatment allocations (mCM or TAU) over a 12-week period.

1 mCM: App-based CM + OAT

Participants in this arm will receive financial incentives contingent on heroin-negative toxicology results. Over a 12-week period, participants will receive thrice-weekly (Monday, Wednesday, Friday) push notifications via the smartphone app when an oral saliva test is due. Smartphones with data plans will be provided to eliminate barriers to access. Participants will be required to conduct an oral saliva test and upload the result within a pre-specified time of receiving the push notification. Oral fluid test kits will be provided to the participants during appointments with the researcher. Participants will receive feedback upon submission, thanking them for uploading their video. The research team will review submissions upon receipt for quality and validity (self-testing and results adequately displayed). Verified heroin-negative tests will result in notification of earnings.

Reinforcer magnitude will be determined by consulting with the PPI group. We anticipate it will be approximately £5 for the first negative test result and escalate by £1 for each subsequent negative test to reach a maximum value of approximately £10. A ‘reset’ procedure will be used, whereby a missed or positive sample will result in a return to base rate for the next negative test. Earnings will be automatically loaded to the app wallet. Participants can access and spend vouchers from multiple vendors. Restrictions on spending categories can be applied, including alcohol, tobacco, and gambling.

The research team will systematically review sleep schedules, work patterns, and other routines with study participants, which could impose constraints on random testing. Schedules will be modified when necessary.

2 TAU: Treatment As Usual

Participants will not receive the mCM intervention and will continue to receive OAT as usual.

### Outcomes

#### Feasibility trial outcomes.

The primary outcome of this feasibility trial is the number of eligible service users recruited over the 6-month recruitment period.

Secondary feasibility outcomes include:

1. The number and percentage of screened service users eligible for inclusion and reasons for ineligibility.
2. The number and percentage of eligible service users who consent to participate in the feasibility trial and the reasons for refusing consent.
3. Adherence to the intervention based on app interactions, responses to push notifications, and uploads.
4. The number/percentage attending follow-up interviews of those randomised.
5. The number/percentage of oral saliva tests uploaded of sufficient quality.
6. The number/percentage of urine samples conducted (collected at four-weekly intervals following randomisation).
7. Acceptability of the intervention among recipients exploring satisfaction and perceived benefits, assessed by qualitative interviews.
8. Acceptability among treatment providers, exploring perceived appropriateness, intent for future adoption, and perceived positive or negative effects on service, assessed by qualitative interviews.

#### Clinical outcomes for a future confirmatory trial.

The primary clinical outcome measure for a future confirmatory trial is the percentage of heroin-negative urine samples (collected at four-week intervals following randomisation).

Secondary outcomes for the future confirmatory trial include:

1. Number/percentage retained in treatment over the 12-week intervention period
2. Opiate Treatment Index (Section 2 – Drug Use) [47]
3. Hospital Anxiety and Depression Scale (HADS) [48]
4. Social functioning measured using the Opiate Treatment Index [47]
5. Stages of Change Readiness and Treatment Eagerness Scale (SOCRATES) [49]
6. Other substance use: heroin and other substances (determined by urine immunoassays)

Items 2–6 will be collected at each assessment timepoint (baseline, weeks 4, 8, 12). We will also collect information needed to inform sample size calculations of the future confirmatory trial:

1. Appropriate summary statistics of the primary clinical outcome.

We will also collect information on:

1. Socio-demographic characteristics (including age, gender, ethnicity, employment status, living situation)

### Participant timeline and study visits

**Fig 1:**
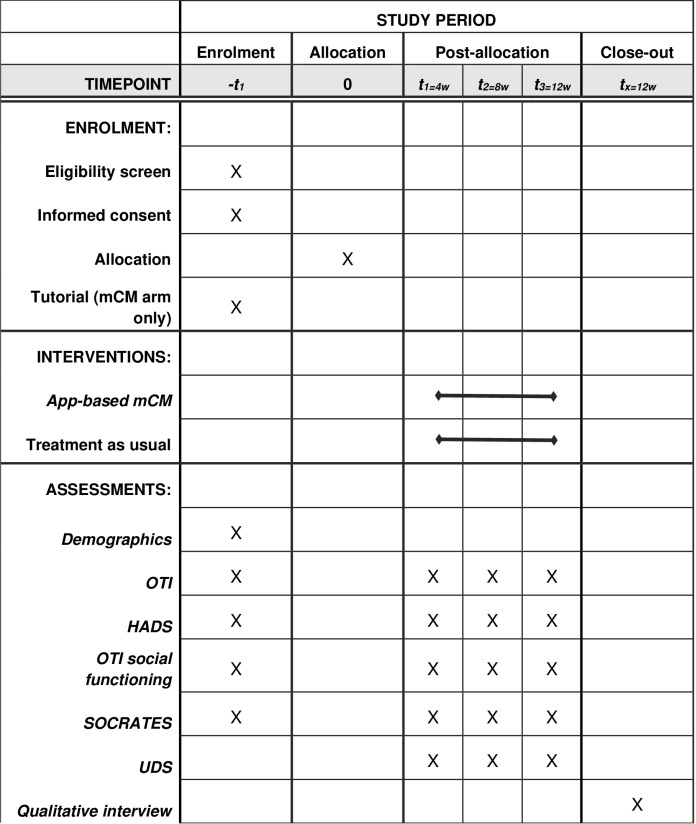
Spirit schedule of enrolment, interventions and assessment.

**Fig 2:**
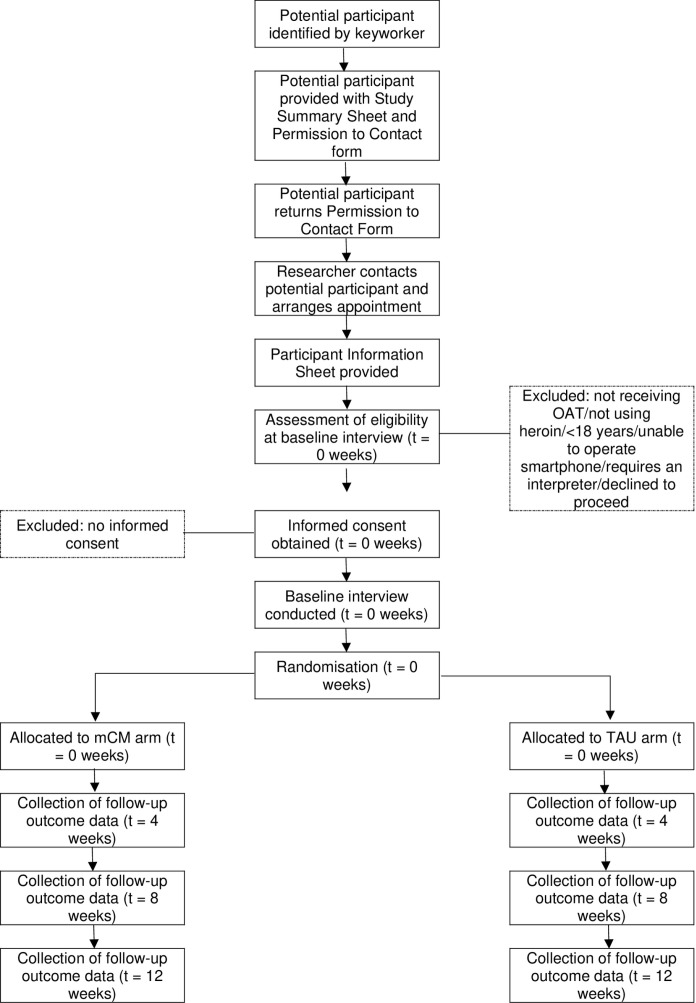
CONSORT diagram illustrating timeline and participant commitment.

Participants will meet with the researcher at four time points: -t1 to complete baseline assessment, randomisation and enrolment and t1, t2 and t3 to provide bio-verified and self-reported assessment on drug use. Participants will be reimbursed £10 each time for their time. Recruitment is projected to take place between 1^st^ July 2025 and 31^st^ December 2025. See SPIRIT schedule of enrolment, interventions and assessments (Fig 1) and CONSORT diagram illustrating timeline and participant commitment (Fig 2).

### Sample size

One of the aims of this feasibility trial is to estimate the parameters needed to inform a sample size calculation for the future confirmatory trial. A sample of 40 was chosen based on the resources available and the number required to estimate feasibility parameters with adequate precision. With 40 participants, we will be able to estimate the expected recruitment rate of 6.7 patients per month (primary feasibility outcome) to within a 95% confidence interval of 4.3 (4.8 to 9.1 patients recruited/month). For the second feasibility outcome (‘Percentage of screened patients eligible for inclusion and reasons for ineligibility’), we will be able to estimate the expected percentage of 50% to within a 95% confidence interval of 42% to 58%. This is based on screening 160 patients, of whom we expect 50% to be eligible, and of these, a half are expected to consent to participate.

### Randomisation

Participants will be randomly assigned to the two groups (mCM and TAU) using Sealed Envelope’s simple randomisation method, ensuring a 1:1 allocation ratio. The randomisation process will be straightforward and will not involve stratification or minimisation due to the small sample size. The details of the randomisation process will be documented and stored in the trial master file.

### Blinding

Due to the nature of the intervention, participants, keyworkers, chief investigator and research team will be unblind to treatment allocation.

### Data collection

There will be four forms of data collection:

1 Smartphone App

The smartphone app will automatically record data on app usage and engagement, including the number of prompts responded to and tests uploaded. The accuracy and reliability of uploads will be measured automatically by the application. At the end of 12 weeks post-enrolment, these data will be extracted from the software system by a researcher and entered into an SPSS database. Usage of the mCM system will be captured through an automated system and any technical problems logged. App usability will be determined using the System Usability Survey [46].

2 Quantitative interviews

Outcome measures will be collected at four time points: baseline and weeks 4, 8, 12. Data will be collected for all participants unless the participant withdraws consent for continued collection of their data.

3 Qualitative interviews

Semi-structured qualitative interviews (analysed thematically) with participants (n = 20) receiving the intervention will assess acceptability and perceived benefits. Interviews will be conducted following the completion of the mCM intervention. They will be audio-recorded and guided by a topic list that is applied flexibly to ensure coverage of key themes while being sensitive to emergent themes.

4 Immunoassay tests

Immunoassays, which use antibodies to detect the presence of specific drugs or metabolites, are the most common method and allow for on-site instant detection of substances. Urine Drug Screen (UDS) using Access Diagnostic Tests multi-drug rapid test kit for qualitative detection of amphetamine (1000ng/ml), benzodiazepines (300ng/ml), cannabis (50ng/ml), cocaine (300ng/ml), methadone (300ng/ml), methamphetamine (1000ng/ml) and opiates (morphine; 2000ng/ml). Test cups also include a urine adulteration test strip for oxidants/specific gravity/pH adulteration test pads and a temperature test strip. Urine samples will be collected at baseline and weeks 4, 8, 12 to determine the primary outcome measure. Results will be available and recorded within 5 minutes. Oral Fluid Test (OFT) using Access Diagnostic Tests panel direct saliva oral fluid drug test kit for qualitative detection of amphetamine (50ng/ml), cannabis (12ng/ml), cocaine (20ng/ml) and opiates (40ng/ml). OFTs will be conducted remotely by participants thrice weekly as part of the CM intervention to determine heroin use. Results will be available within 5 minutes. All used equipment will be discarded immediately after use.

### Data analysis

Data analysis will be conducted by the study chief investigator (CAG). A statistical analysis plan will be developed and agreed with the trial steering committee (see below). All quantitative data will be analysed using SPSS or R. Feasibility and clinical outcomes will be summarised using appropriate statistics, e.g., mean/standard deviation or median/interquartile range for continuous variables and frequencies and percentages for categorical variables. Statistical analyses will not be powered to estimate the efficacy of the intervention (i.e., differences between arms). Estimates of treatment effects will be treated as exploratory and not used to make inferential statements. Progression to a larger confirmatory trial will be assessed based on pre-specified progression criteria (see section 3.17).

Clinical outcomes for the future confirmatory trial will also be summarised using appropriate statistics (as above). Differences between arms will be summarised (e.g., differences in means or percentages) but not used as the basis for inferential statements. The primary purpose of these estimates is to inform sample size calculations for a future confirmatory trial. This analysis is not powered to detect differences between arms. Estimates of treatment effect will be treated as exploratory and not used as the basis for inferential statements. Analyses will be done under the intention-to-treat principle: there will be no per-protocol or subgroup analyses.

All efforts will be made to avoid missing baseline data (i.e., requiring completion of baseline data before randomisation), but if this occurs, missing values will be imputed according to current recommendations. Missing scale item data will be handled as per questionnaire-specific recommendations, or if no recommendations exist, using prorating (if less than 20% of the items are missing for a given individual, the missing items will be replaced by the mean of their complete items). Given this is a feasibility study and the focus is not on between-arm comparisons, multiple imputation for missing data will not be used.

Qualitative interviews will be transcribed verbatim and subject to a thematic analysis. After familiarisation with the data, an initial coding frame will be developed, built upon from both the a priori topic guide and themes developed in the data. This coding frame will be developed and refined as data collection and analysis progress. The analytical stage will seek to discern patterns, consistencies, and divergences in the data and support the identification of themes that enable a comprehensive and detailed response to the research questions.

There are no interim analyses or audits of trial conduct planned.

### Data monitoring

A trial steering committee (TSC) will be formed before the start of recruitment consisting of an independent chair, at least two independent members and members of the research team (as non-voting members). A charter will be written and agreed upon by members outlining the remit and functioning of the TSC, including meeting frequency. The TSC will review recruitment data (and compare to pre-specified targets) and information about participant characteristics and safety (adverse events). Data will be summarised and not split by arm. Therefore, we will form an independent executive committee to monitor safety during the trial. Before each TSC meeting, the executive committee will review the number of adverse events and serious adverse events by arm and make recommendations in writing to the TSC chair (e.g., if the committee feels the trial needs to be stopped). The TSC chair would then raise these issues for discussion and decision by the TSC.

### Data protection

Researchers will collect data from the baseline and follow-up interviews on paper case report forms. These forms will be stored securely at King’s College London and entered into an SPSS database. Data stored in the trial database will include participant’s unique PIN number, initials and date of birth. The trial database will only be accessible to the chief investigator. Data will be stored in password protected KCL network. At the point of giving consent, participants will be told how their personal data is going to be used, how it will be stored, and for how long. The trial data will be registered on the King’s Data Protection Registration.

App developers protect data through advanced network segmentation, firewalls, encryption, and real-time threat monitoring. They use Amazon Shield and WAF to block cyberattacks, while AWS Network Firewall and Route 53 secure network traffic. Encryption and key management services like AWS KMS and Secret Manager safeguard sensitive information. CrowdStrike security solutions provide 24/7 threat detection, ensuring rapid response to cyber threats, ransomware, and data leaks. For authentication, Auth0 and AWS enable secure and scalable identity management, allowing seamless integration with customer identity providers. Together, these measures ensure comprehensive security across the developer’s infrastructure. Data will be retained for a period of 2 years after the completion of the research study to allow for data validation and archival purposes. During this time, the data will be anonymised, and no personal identifiers will be retained. Data will not be used for any commercial purposes.

### Adverse event monitoring

We will monitor non-serious adverse events, serious adverse events, serious adverse reactions to trial interventions, serious deterioration, and active withdrawals from treatment. Keyworkers will be asked to record (on a case report form) and notify us if they are aware of any adverse events or active withdrawals from treatment. We will contact key workers once a week to monitor possible adverse reactions. These will be recorded in a specific SPSS database, stored on a secure King’s College London drive and reported to the independent executive committee.

Keyworkers will be asked to record any adverse events. Any Serious Adverse Events should be reported to the PI within 24 hours. Any Serious Adverse Events related to the intervention will be reported to the Research Ethics Committee within 15 days.

### End of study

The end of the study refers to the point at which trial databases are locked for analysis.

### Progression criteria

Progression to a larger confirmatory trial will be evaluated based on the pre-specified progression criteria (see Table 1). However, not achieving ‘Green’ does not necessarily indicate the unfeasibility of a future trial but would indicate that changes are needed to recruitment procedures, attendance record keeping and follow-up resources.

**Table 1:** Pre-specified progression criteria.

	Green	Amber	Red
Number of eligible patients enrolled over the 6-month recruitment period	≥30, at least 15 per arm	≥20, at least 10/arm	<20
Percentage of screened patients who are eligible for inclusion in the feasibility trial.	≥50%;	≥30%;	<30%
Adherence to the intervention based on percentage of requested videos uploaded	≥70% in CM arm	≥50% in CM arm	<50% in CM arm
Percentage of urine drug screenings completed, overall	≥70% in all arms	≥50% in all arms	<50% in all arms
Number and percentage attending follow-up interview at end of 12-week intervention period (compared to number randomised)	≥70%	≥50%	<50%

### Dissemination

We will provide a plain English summary of our findings to patients participating in the study. We will also provide summaries to drug services and via SLaM NHS Trust. We will publish our findings in open-access peer-reviewed journals.

## Discussion

Research on Contingency Management has grown exponentially over the last decade, with remote technologies being leveraged more than ever to expand the reach and scope of these interventions [24]. This coincides with technological innovations in the delivery of healthcare interventions in general but intensified due to the COVID-19 pandemic and public health control measures [50,51]. Digital CM has the potential to expand the scope of CM delivery, targeting behaviours that occur outside the clinical setting and overcoming the known barriers to CM implementation. Existing evidence supports the feasibility and effectiveness of digital CM to address a range of treatment-related behaviours [25–32,34–41] and high levels of acceptability towards the remote monitoring of behaviour and delivery of reinforcement among service user recipients [42,43] and non-recipients [44]. However, there is yet to be a fully remote CM intervention targeting heroin abstinence among an opioid treatment population.

This study will involve the development of a novel and advanced smartphone app that can fully digitalise a CM intervention to monitor frequent heroin use and provide incentives for results consistent with treatment goals. Developing and delivering a fully remote mCM intervention for reducing heroin use among individuals in treatment for OUD holds immense potential. This novel approach can significantly broaden the scope of CM treatment, providing a more comprehensive and accessible solution for individuals with opioid use disorder.

## Supporting information

S1 Protocol Detailed protocol. (PDF)

S1 Checklist SPIRIT checklist. (DOCX)
