# Supplementary material for: Mobile telephone-delivered Contingency Management (mCM) to reduce heroin use in individuals with opioid use disorder (CM4OUD): A feasibility study protocol
Source: PLoS One. 2025 May 28;20(5):e0324516. doi: 10.1371/journal.pone.0324516 (PMC12118896; doi:10.1371/journal.pone.0324516)
Supplement: S1 Protocol — (PDF) [file pone.0324516.s001.pdf]

Protocol

Mobile telephone-delivered Contingency Management (mCM) to reduce heroin use in individuals with opioid use disorder (CM4OUD)

Version 1.2 08.01.25

Funded by:

Society for the Study of Addiction (SSA)

Sponsor:

South London & Maudsley NHS Foundation Trust & King’s College London

Trial Registration: ISRCTN 60538178

Date of registration: 02.12.24

Table of Contents

1 General Information 3
1.1 Sponsor 3
1.2 Funder 3
1.3 Chief Investigator 3
1.4 Research team 4
1.5 Collaborators 4
1.6 Trial Steering Committee 4
2 Background and rationale 4
3 Aims & Objectives 5
4 Theoretical Framework 5
5 Smartphone App 6
6 Workstream 1: Design, develop and test the intervention (Pilot study) 6
6.1 Aims and objectives 6
6.2 Methodology 6
6.2.1 Design 6
6.2.2 Participants 7
6.2.3 Data collection 7
6.2.4 Data analysis 8
7 Workstream 2: Feasibility trial 8
7.1 Aims and objectives 8
7.2 Methodology 8
7.2.1 Participants 8
7.2.2 Design 9
7.2.3 Intervention 9
7.2.4 Sites 9
7.3 Eligibility criteria 10
7.4 Outcomes 10
7.4.1 Feasibility trial outcomes 10
7.4.2 Clinical outcomes for a future confirmatory trial 10
7.5 Sample size 11
7.6 Recruitment 11
7.6.1 Screening 11
7.6.2 Consent 12

7.6.3 Enrolment ... 12
7.6.4 Withdrawal procedures ... 13
7.7 Data collection ... 13
7.8 Data analysis ... 14
7.9 Participant timeline ... 15
7.10 Randomisation ... 16
7.11 Blinding ... 16
7.12 App security ... 16
7.13 Monitoring ... 17
7.14 Adverse events ... 18
7.14.1 Definitions ... 18
7.14.2 Adverse event reporting ... 19
7.15 GDPR compliance ... 19
7.16 Data management ... 19
7.17 End of study ... 20
7.18 Dissemination, Outputs & Impact ... 20
7.19 Intellectual Property ... 20
7.20 Progression criteria ... 20
8 Workstream 3: Experience Sampling Methodology (ESM) ... 21
8.1 Aims and objectives ... 21
8.2 Methodology ... 21
8.2.1 Design ... 21
8.2.2 Participants ... 22
8.2.3 Data collection ... 22
8.2.4 Data analysis ... 22
9 Project timeline ... 22
10 Project management ... 23
11 Ethics/Regulatory Approvals ... 23
12 Protocol amendments ... 23
13 References ... 24

1 General Information

1.1 Sponsor

Professor Bashir Al-Hashimi	Vice President (Research and Innovation)<br>King's College London<br>Room 8.11, 8th Floor Melbourne House<br>44-46 Aldwych<br>London WC2B 4LL<br>Tel: 02078487306<br>Email vpri@kcl.ac.uk (cc Susan.Dickson@kcl.ac.uk)
Mrs Abnash Kaur Chauhan	South London and Maudsley NHS Foundation Trust<br>R&D Department<br>Room W1.08<br>Institute of Psychiatry, Psychology & Neuroscience (IoPPN)<br>De Crespigny Park<br>London SE5 8AF<br>020 7848 0339<br>slam-ioppn.research@kcl.ac.uk

The sponsor has overall responsibility for proportionate, effective arrangements being in place to set up, run and report the research project. The sponsor has overall responsibility for the research as set out in the UK Policy Framework of Health and Social Care 2017 (para 9.10).

1.2 Funder

The Society for the Study of Addiction has funded this project as part of an Academic Fellowship awarded to Dr Carol-Ann Getty (PI).

1.3 Chief Investigator

Dr Carol-Ann Getty (PI)	King's College London and South London and Maudsley NHS Foundation Trust (Lead researcher and has responsibility for its overall conduct)

1.4 Research team

Dr Nicola Metrebian	Primary mentor	King's College London
Professor Sir John Strang	Secondary mentor	King's College London
Dr Ewan Carr	Statistics mentor	King's College London
Dr Mike Kelleher	SLaM PI	Lorraine Hewitt House

1.5 Collaborators

Contingency Management Innovations (CMI)	Software company

1.6 Trial Steering Committee

TBC	TBC

2 Background and rationale

Opioid use disorder is a major public health issue. Deaths globally from opioid overdose continue to rise, exceeding 100,000 annually (1). Recovery from opioid use disorder is a long-term and complex process, with the average time in treatment for opiate problems around 2 years longer than for other substances (2). Half of adults receiving treatment in the UK for substance use are there for problems with opiates, and of these, only a quarter are discharged as 'treatment completed' (2). The persistent prevalence of heroin use disorders necessitates comprehensive and innovative interventions to address this complex issue.

Contingency Management (CM), where positive reinforcement (i.e., rewards or incentives) is delivered upon evidence of positive behaviour change, is among the most efficacious psychosocial interventions for substance use disorder (SUD). Meta-analyses have found CM to reduce illicit substance use and promote treatment-related behaviours such as retention in treatment ((3-8). CM targeting opioid abstinence in service users undergoing treatment for opioid use disorder (OUD) was associated with a medium-large effect size on abstinence versus a control intervention (Cohen's d = 0.58; 95% CI, 0.30-0.86; I2 = 75.9%) (9).

Despite NICE recommendations to integrate CM into addiction services (10), the UK has a limited track record of applying these interventions. One way to dramatically increase access to CM while minimising the burden on resources and staff is to deliver them remotely using mobile phones. Innovations in technology enable CM to be implemented with enhanced fidelity and reduced cost without compromising effectiveness (11, 12). Remote technologies allow CM to target behaviours that occur outside the clinic. Mobile telephones can deliver prompts and be used to upload video/photo evidence of target behaviours (e.g., breath carbon monoxide (CO) or alcohol test to verify smoking/alcohol abstinence). Immediate positive reinforcement can be delivered remotely (12).

Previous work undertaken by the research team found mobile-delivered CM (mCM) to be effective in increasing smoking and alcohol cessation (13). High levels of acceptability towards remote monitoring of behaviour and delivery of reinforcement among service user recipients (14) and non-recipients (15) were also found, with 81% reporting to be in favour of incentive programmes. Importantly, 96% of service users owned a mobile telephone (85% of which were smartphones), suggesting it is a viable approach. Consultations with policy and clinical stakeholders have provided further support for the development and implementation of these interventions.

However, a significant gap exists in the realm of opioid use. Currently, there are no mCM interventions specifically targeting illicit opioid use among individuals undergoing treatment for OUD. This represents an untapped opportunity to expand the scope of CM treatment for OUD, potentially revolutionising the landscape of addiction interventions. The absence of mCM interventions targeting illicit opioid use highlights a critical need for innovation in addressing this complex aspect of substance use. Developing and delivering a fully remote mCM intervention for reducing opioid use among individuals in treatment for OUD holds immense potential. This novel approach has the capacity to significantly broaden the scope of CM treatment, providing a more comprehensive and accessible solution for individuals with opioid use disorder.

3 Aims & Objectives

This project aims to (1) develop and test a mobile telephone-delivered Contingency Management (mCM) intervention to reduce opioid use in individuals with opioid use disorder; (2) undertake a trial to determine the feasibility of a larger confirmatory trial of clinical effectiveness, and (3) investigate the dynamic interplay of psychological, environmental, contextual factors and the mCM intervention on influencing heroin use in real-time.

To achieve these aims, the study will consist of three interlinked workstreams as described in this protocol:

1. Design, develop and test
2. Feasibility trial
3. Experience Sampling Methodology (ESM)

4 Theoretical Framework

A systematic and evidence-based approach will be adopted, integrating the ‘Behavioural Change Wheel’ (16) and ‘Developing Digital Interventions: A Methodological Guide’ (17). This approach will consider behaviour change theories, user input, and iterative testing throughout the development process.

The *theory and evidence-based* approach is centred on the ‘Behaviour Change Wheel (BCW)’ with consideration of the ‘Developing Digital Interventions: A Methodological Guide’. Integrating these frameworks will ensure the systematic alignment of the intervention with established behaviour change theories, enhancing the potential for successful outcomes. The stages of development are outlined in Appendix A.

5 Smartphone App

The mCM smartphone app will be designed and developed in collaboration with a digital health company, ‘Contingency Management Innovations (CMI)’. CMI is an industry-leading developer and supplier of technology for designing, implementing, and securely managing CM programs. CMI has developed Contingency Management platforms in conjunction with the National Institutes of Health (NIH) and deployed its solutions in various commercial settings, including large state-level rollouts.

Janus technology, the incentive management platform utilised by healthcare institutions, enables the design, customisation, deployment, and administration of CM programs in a secure, efficient, and impactful manner. Developed by CMI, the Janus technology incorporates a versatile CM engine capable of tailoring budget management, reinforcement scheduling, and the targeting of diverse events and behaviours through configurable reset mechanisms. Janus offers a clinician dashboard, enabling healthcare professionals to monitor and track their client’s progress.

6 Workstream 1: Design, develop and test the intervention (Pilot study)

6.1 Aims and objectives

The aims of Workstream 1 are as follows:

1. Design and develop a new behavioural intervention to remotely monitor opioid use and deliver financial incentives for verified heroin abstinence.
2. Investigate the usability and functioning of a newly developed mCM intervention to monitor heroin use.

Objectives:

3. Evaluate approaches to objective monitoring of heroin use (e.g., oral fluid tests/urinalysis);
4. Determine intervention specifics such as frequency of monitoring behaviour, preferred reinforcer and reinforcement schedule;
5. Develop a prototype smartphone app to host the mCM intervention;
6. Determine the reliability and operationalisation of the intervention (captured automatically via the app);
7. Assess participant adherence to intervention procedures;
8. Determine usability using the System Usability Survey (18);
9. Detect and resolve technical problems.

6.2 Methodology

6.2.1 Design

A small-scale pilot test will be undertaken to observe real-world usage and identify any unforeseen issues. This testing phase will be carried out by service users undergoing treatment for opioid use disorder in UK addiction services (n=5-10) over one week. During this time, participants will receive the intervention (respond to sample requests, conduct testing, and upload videos). Feedback will be gathered for further refinement before

progressing to the feasibility study (Workstream 2). An inclusive and collaborative approach involving key stakeholders will be adopted, whereby researchers and developers work together with client and treatment provider stakeholders to create solutions that meet the needs, preferences, and contexts of the intended users. It emphasises active participation, shared decision-making, and iterative feedback loops. The active involvement of users and stakeholders throughout the process increases the app's relevance and usability in real-world settings.

The formal testing phase will include the following stages:

1. **Induction & testing**
    a. Participants will receive a tutorial of the mCM app.
    b. Once inducted, participants will be enrolled on the system and provided with instructions on participation.
    c. Participants will undergo a one-week testing period, engaging with the mCM intervention, responding to sample request push notifications, conducting testing and uploading videos.
2. **Feedback & refine**
    a. After the testing period, participants will engage in focus groups, exploring usability, acceptability, reliability and functionality of the app.
    b. The BCW will be used to ensure alignment with behaviour change principles.
    c. Participant feedback will aid app refinement.
3. **Finalise**:
    a. Following final iterations, focus groups will be repeated, employing think-aloud techniques to assess perceptions and interactions.
    b. If necessary, the app will be modified to enhance user experience and adherence to behaviour change principles.

6.2.2 Participants

Participants (n=5-10) will be service users undergoing treatment for opioid use disorder in UK addiction services. Participants will be recruited using a convenience sampling approach, whereby treatment providers will identify actively engaged service users who might be willing to participate. Due to the intensity of this testing phase, it is important to minimise attrition and maximise involvement. Therefore, service users approached will be those stabilised on opioid agonist treatment with the capability of fulfilling testing requirements. Participants will be provided with a Participant Information Sheet and asked to provide informed consent before participating.

6.2.3 Data collection

Participants will be asked to keep a diary of their experiences using the app to overcome problems of poor recall. Participants will be asked to note any aspects that they found particularly useful or not useful, easy to use or problematic and aspects which they liked or disliked. Focus groups will also be conducted to enable participants to share their thoughts and reactions as they interact with the mCM app. During the focus group sessions, participants will be prompted to articulate their perceptions, feelings, and decision-making processes in real-time, providing valuable insight into their user experience. By verbalising their thoughts aloud, participants provide researchers with insight into their cognitive

processes and the factors influencing their interactions with the app, such as usability, acceptability, and functionality. Employing the Behaviour Change Wheel (BCW) framework ensures alignment with behaviour change principles, guiding the refinement of the digital intervention to enhance user engagement and adherence.

The smartphone app will automatically record participant usage and engagement data, including the number of prompts responded to and test uploads. Technological issues will be detected.

6.2.4 Data analysis

Focus groups will be analysed using Thematic Analysis, as developed by Braun and Clarke (2006). This will allow us to identify participants' experiences with the mCM app, perspectives on its usability and acceptability, and any problems or barriers.

7 Workstream 2: Feasibility trial

7.1 Aims and objectives

This study aims to determine the acceptability and feasibility of conducting a future randomised controlled trial of the clinical effectiveness of mobile telephone-delivered Contingency Management (mCM) to encourage heroin abstinence.

Feasibility study objectives are as follows:

Primary objective:

1. Determine the number of eligible service users and recruitment rates.

Other objectives are to:

2. Determine adherence to the intervention (e.g., user engagement, responses to prompts, uploads);
3. Determine follow-up rates at 12 weeks;
4. Explore intervention acceptability among participants (including those not receiving the mCM intervention) and treatment providers;
5. Determine usability using the System Usability Survey (18);
6. Explore the suitability and participant acceptability of alternative measures for the primary clinical outcomes;
7. Characterise aspects of the primary outcome needed for a sample size calculation for a larger trial (including an estimate of the intraclass correlation).

7.2 Methodology

7.2.1 Participants

We will target the clinically significant group of existing patients in UK addiction services, who are receiving opioid agonist treatment for opioid use disorder and continue to use heroin.

7.2.2 Design

The feasibility study will use an individually randomised controlled design, where service users will be recruited and randomly assigned (1:1) to one of the conditions below over a 12-week period:

A. Mobile Contingency Management (mCM): Smartphone App plus incentives for objective verification of heroin abstinence
B. Treatment As Usual (TAU)

The trial design has been created to reflect the future confirmatory trial in which the novel intervention "Mobile Contingency Management" (mCM; Arm A) will be compared to a control condition (TAU; Arm B) in which no Contingency Management will be received.

7.2.3 Intervention

Each participant will be randomly allocated (1:1) to one of two treatment allocations (mCM or TAU). All participants will continue to receive OAT as part of their treatment for OUD.

1. Mobile Contingency Management (mCM):

Participants in this arm will receive financial incentives contingent on heroin-negative drug samples. Over a 12-week period, participants will receive thrice-weekly push notifications via the smartphone app when an oral saliva test is due. Participants will be required to conduct an oral saliva test and upload the result within 90 minutes of receiving the push notification. Oral fluid tests will be provided to the participants during appointments with the researcher. Participants will receive feedback upon submission, thanking them for uploading their test result. The research team will review submissions upon receipt for quality and validity (self-testing and results adequately displayed). Verified heroin-negative tests will result in notification of earnings.

Earnings will start at £2 for the first negative test result and escalate by £1 to reach a maximum value of £5 after four consecutive negative tests. A 'reset' procedure will be used, whereby a missed or positive sample will result in a return to £2 for the next negative test.

Earnings will be automatically loaded to the app wallet. Participants can access and spend vouchers from multiple vendors. Restrictions on spending categories will be applied, including alcohol, tobacco, and gambling. Participants could earn a maximum of £174 over the 12-week study period.

The research team will systematically review sleep schedules, work patterns, and other routines with study participants, which could impose constraints on random testing. Schedules will be modified when necessary.

2. TAU: Treatment As Usual

Participants will not receive the mCM intervention and will continue to receive treatment as usual.

7.2.4 Sites

A 'site' is a drug service providing treatment to individuals with substance use disorder. Two sites will be recruited: one from South London and the Maudsley NHS Foundation Trust

(SLaM NHS) and one from a non-NHS service. Drug services will be eligible if they provide treatment to individuals with opioid use disorder.

7.3 Eligibility criteria

The eligibility criteria for enrolling service user participants will include:

1. Receiving opioid agonist treatment (methadone or buprenorphine, including pro-longed release);
2. Self-reported heroin use at least 1 day/week;
3. Aged ≥ 18 years;
4. Able to operate an Android or iOS smartphone with acceptable capability;
5. Willing to receive 12-week CM intervention;
6. Able to provide informed consent.

Service users will be excluded if participating in any other research studies.

7.4 Outcomes

7.4.1 Feasibility trial outcomes

The primary outcome of this feasibility trial is the number of eligible service users recruited over the 6-month recruitment period.

Secondary feasibility outcomes include:

1. The number and percentage of screened service users eligible for inclusion and reasons for ineligibility.
2. The number and percentage of eligible service users who consent to participate in the feasibility trial and the reasons for refusing consent.
3. Adherence to the intervention based on app interactions, responses to push notifications, and uploads.
4. The number/percentage attending follow-up interviews of those randomised.
5. The number/percentage of oral saliva tests uploaded of sufficient quality.
6. The number/percentage of urine samples conducted (collected at four-weekly intervals following randomisation).
7. Acceptability of the intervention among recipients exploring satisfaction and perceived benefits, assessed by qualitative interviews.
8. Acceptability among treatment providers, exploring perceived appropriateness, intent for future adoption, and perceived positive or negative effects on service, assessed by qualitative interviews.

7.4.2 Clinical outcomes for a future confirmatory trial

The primary clinical outcome measure for a future confirmatory trial is the percentage of heroin-negative urine samples (collected at four-week intervals following randomisation).

Secondary outcomes for the future confirmatory trial include:

1. Number/percentage retained in treatment over the 12-week intervention period
2. Opiate Treatment Index (Section 2 - Drug Use) (19)
3. Hospital Anxiety and Depression Scale (HADS) (20)
4. Social functioning measured using the Opiate Treatment Index (19)
5. Stages of Change Readiness and Treatment Eagerness Scale (SOCRATES) (21)
6. Other substance use: opioid and non-opioid (determine by urine immunoassays)

Items 2-6 will be collected at each assessment timepoint (baseline, week 4, 8 and 12). We will also collect information needed to inform sample size calculations of the future confirmatory trial:

9. Appropriate summary statistics of the primary clinical outcome.

We will also collect information on:

10. Socio-demographic characteristics (including age, gender, ethnicity, employment status, living situation)

7.5 Sample size

One of the aims of this feasibility trial is to estimate the parameters needed to inform a sample size calculation for the future confirmatory trial. A sample of 40 was chosen based on the resources available and the number required to estimate feasibility parameters with adequate precision. With 40 participants, we will be able to estimate the expected recruitment rate of 6.7 patients per month (primary feasibility outcome) to within a 95% confidence interval of 4.3 (4.8 to 9.1 patients recruited/month). For the second feasibility outcome (‘Percentage of screened patients eligible for inclusion and reasons for ineligibility’), we will be able to estimate the expected percentage of 50% to within a 95% confidence interval of 42% to 58%. This is based on screening 160 patients, of whom we expect 50% to be eligible, and of these, a half are expected to consent to participate.

7.6 Recruitment

Over a 6-month period, we aim to recruit 40 service users receiving OAT who continue to use heroin.

7.6.1 Screening

Individuals receiving OAT and continuing to use heroin will be identified by their keyworker at participating clinics. Potential participants will be provided with the Study Summary Sheet and asked to sign the Permission to Contact form. The researcher will contact those service users and arrange an appointment. During this appointment, willing service users will be screened for eligibility and, if eligible, provided with the Participant Information Sheet.

Reasons for ineligibility for the trial will be recorded. Eligibility logs will be kept at each site. No personally identifiable information will be recorded on these forms. Each will be provided with a unique sequential number. Screening forms of those not enrolled will be entered into a separate database. These will be completely anonymous. No identifying information will be recorded. Researchers will complete the Screening Log, which will be kept in the Trial Investigators’ File. All completed screening forms will be stored in the study Site Trial File.

This will be taken back to the Addictions Department at the IoPPN, KCL and stored in a locked filing cabinet.

Potential participants will be informed that:

- The study will test the feasibility of a future trial of a new smartphone app delivering incentives to encourage abstinence from heroin.
- They will be randomised to one of two treatment options (mCM or TAU).
- If assigned to the mCM arm, they will be provided with a study smartphone and need to maintain access to the app daily to ensure push notifications can be received and responded to in a timely manner.
- Their participation in the trial will not affect their treatment, i.e., if they do not participate in the trial, they will still be provided with a named keyworker and receive treatment as usual.
- Treatment providers will be able to view their progress, including test results.
- They will complete a research interview and urine drug test at several timepoints: baseline, 4 weeks, 8 weeks, and 12 weeks. These appointments will last approximately 1 hour, and they will receive a £10 voucher each time for their time and travel.

7.6.2 Consent

Once screened, eligible service users will be provided with information about the research and asked if they would like to participate. If willing to take part, patients will be asked to provide informed written consent. Patients will be asked to make an immediate decision about their participation in the study. The researcher will obtain consent during the appointment. Although patients are given the study information sheet and asked to consent to the study at the first appointment, they can withdraw their consent at any point throughout the study.

Patients will be asked to sign the participant Consent Form. Signed copies of which will be given to the participant and filed in the patient’s notes. Consent forms will be brought back to the Addictions Department, IoPPN, KCL and stored in a locked filing cabinet.

The recruitment procedures described above conform with standard requirements that a responsible clinician (in this case, the participant’s drug treatment assessment nurse/drug worker) invites the patients to participate in the research. All willing patients will have to provide Permission to Contact before being contacted by the researcher and provided with more information. Potential participants will be seen by a researcher within one week. Hence, researchers will not have contact with patients (or have access to data about them) without patients first providing Permission to Contact.

7.6.3 Enrolment

The baseline interview will be conducted before the researcher informs the participant what treatment arm they have been allocated to and provides the specific information accordingly:

<u>Mobile Contingency Management (mCM)</u>

Participants randomised to the mCM condition will be informed that:

- They will receive a study smartphone with the mCM app installed. They must ensure this phone is fully operational at all times.
- They will receive push notifications at random times (thrice weekly) with specific instructions (e.g., to carry out an oral saliva swap test and upload the test results). The result will be expected within 90 minutes of receiving the push notification.
- Sleep schedules, work patterns and other routines that could impose constraints on random testing will be considered.
- Feedback will be received upon submission. The research team will review submissions upon receipt for quality and validity (self-testing and results adequately displayed).
- Verified heroin-negative tests will result in notification of earnings. Earnings will start at £2 for the first negative test result and escalate by £1 to reach a maximum value of £5 after four consecutive negative tests. A 'reset' procedure will be used, whereby a missed or positive sample will result in a return to £2 for the next negative test.
- Earnings will be automatically loaded to the app wallet. Participants can access and spend vouchers from multiple vendors. Over the 12-week study period, participants could earn a maximum of £174 in incentives.
- Their healthcare provider will be able to track their progress.
- Research interviews and urine drug testing will be conducted at several timepoints: baseline, 4 weeks, 8 weeks, and 12 weeks. These appointments will last approximately 1 hour, and they will receive a £10 voucher each time for their time and travel.

<u>Treatment as usual (TAU)</u>

Participants randomised to the TAU condition will be informed that:

- They will continue to receive their treatment as usual.
- Research interviews and urine drug testing will be conducted at several timepoints: baseline, 4 weeks, 8 weeks, and 12 weeks. These appointments will last approximately 1 hour, and they will receive a £10 voucher each time for their time and travel.

The researcher (CAG) will then enrol the participant onto the system. Keyworkers will be informed (by the researcher) of which of their service users are taking part in the study. They will also receive instructions on how to access the healthcare provider platform.

The researcher will complete the Screening Log and Patient Identification Log/Enrolment Log, which will be kept in the Site Trial File.

7.6.4 Withdrawal procedures

Participants are free to withdraw from the study at any time without giving a reason. Withdrawn participants will no longer receive the intervention. However, we will still aim to collect outcome data from these participants.

7.7 Data collection

There will be four forms of data collection:

Smartphone App

The smartphone app will automatically record data on participant usage and engagement, including number of prompts responded to and test uploads. The accuracy and reliability of uploads will be measured automatically by the application. At the end of 12 weeks post-enrolment, these data will be extracted from the software system by a researcher and entered into an SPSS database. This will be stored with other trial databases in password-protected files on a King's College London (KCL) secure network drive.

Usage of the mCM system will be captured through an automated system and any technical problems logged. App usability will be determined using the System Usability Survey.

Quantitative interviews

The researcher will conduct face-to-face interviews with participants at baseline (before enrolment) and 12 weeks post-enrolment. Data will be collected for all participants unless the participant withdraws consent for continued collection of their data.

Qualitative interviews

Semi-structured qualitative interviews (analysed thematically) with participants receiving the intervention will assess acceptability and perceived benefits. Interviews will be conducted following the completion of the mCM intervention. They will be audio-recorded and guided by a topic list that is applied flexibly to ensure coverage of key themes while being sensitive to emergent themes.

Drug testing

Oral saliva drug testing will be immediate and any used equipment will be discarded immediately after use. No saliva samples will be stored or analysed further. Urine samples will be collected from study participants at several time points (baseline, weeks 4, 8 and 12) to determine primary outcome measure. Immunoassays, which use antibodies to detect the presence of specific drugs or metabolites, are the most common method and allow for on-site instant detection of substances. Samples will be collected in an all-in-one test kit and results displayed within several minutes. Samples will be disposed of afterwards.

7.8 Data analysis

Data analysis will be conducted by study PI (CAG). A statistical analysis plan will be developed and agreed with the trial steering committee (see below). All quantitative data will be analysed using SPSS or R. Feasibility and clinical outcomes will be summarised using appropriate statistics e.g., mean/standard deviation or median/interquartile range for continuous variables; frequencies and percentages for categorical variables. Statistical analyses will not be powered to estimate efficacy of the intervention (i.e., differences between arms). Estimates of treatment effects will be treated as exploratory and not used to make inferential statements. Progression to a larger confirmatory trial will be assessed based on pre-specified progression criteria (see below).

Clinical outcomes for the future confirmatory trial will also be summarised using appropriate statistics (as above). Differences between arms will be summarised (e.g., differences in means or percentages) but not used as the basis for inferential statements. The primary purpose of these estimates is to inform sample size calculations for a future confirmatory

trial. This analysis is not powered to detect differences between arms. Estimates of treatment effect will be treated as exploratory and not used as the basis for inferential statements. Analyses will be done under the intention-to-treat principle; there will be no per-protocol or subgroup analyses.

All efforts will be made to avoid missing baseline data (i.e., requiring completion of baseline data before randomisation), but if this occurs, missing values will be imputed according to current recommendations. Missing scale item data will be handled as per questionnaire-specific recommendations, or if no recommendations exist, using prorating (if less than 20% of the items are missing for a given individual, the missing items will be replaced by the mean of their complete items). Given this is a feasibility study and the focus is not on between-arm comparisons, multiple imputation for missing data will not be used.

Qualitative interviews with participants receiving the intervention will assess acceptability and perceived benefits. Interviews will be transcribed verbatim and subject to a thematic analysis. After familiarisation with the data (reading transcripts), an initial coding frame will be developed, built upon both the a priori topic guide and themes developed in the data. This coding frame will be developed and refined as data collection and analysis progress. The analytical stage will seek to discern patterns, consistencies and divergences in the data and to support the identification of themes that enable a comprehensive and detailed response to the research questions.

7.9 Participant timeline

Overall schedule and time commitment for trial participants are illustrated in Figure 1.

Figure 1: Schematic diagram

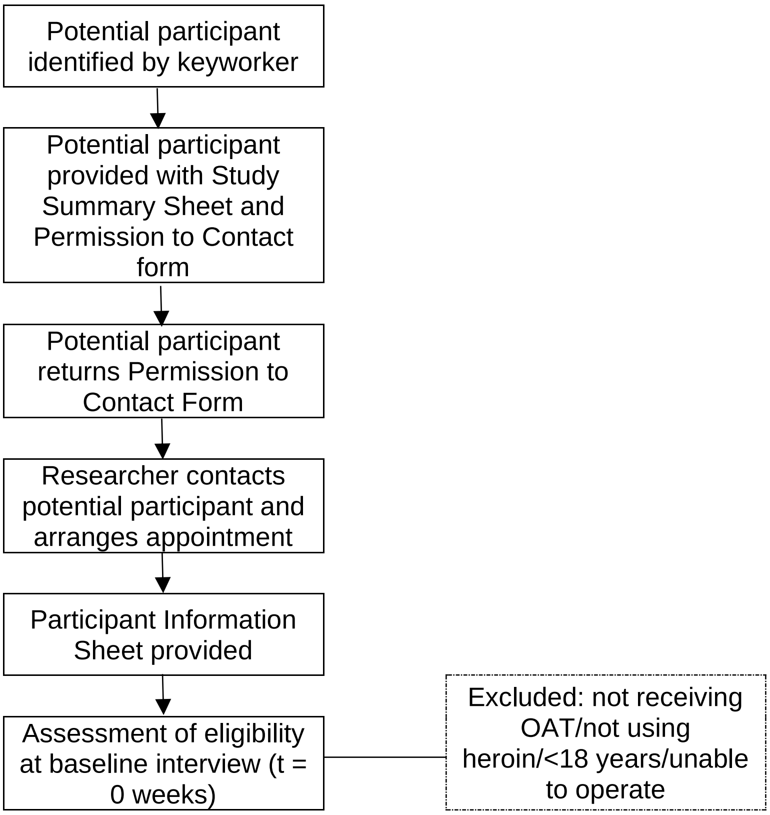

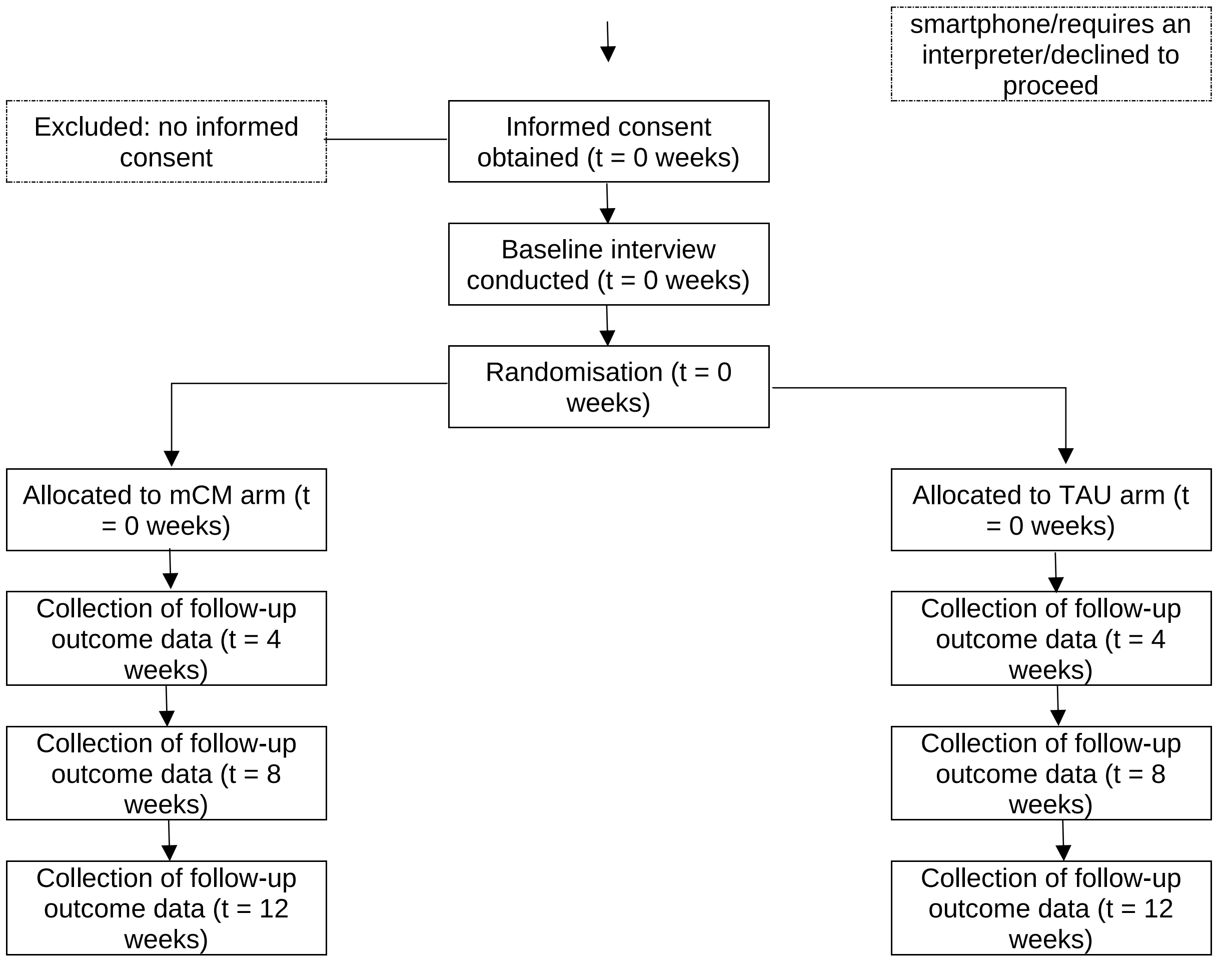

7.10 Randomisation

Participants will be randomly assigned to the two groups (mCM and TAU) using Sealed Envelope's simple randomisation method, ensuring a 1:1 allocation ratio. The randomisation process will be straightforward and will not involve stratification or minimisation due to the small sample size. The details of the randomisation process will be documented and stored in the trial master file (TMF).

7.11 Blinding

The participants, keyworkers, Chief Investigator and research team will be unblind to treatment allocation due to the nature of the intervention.

7.12 App security

CMI minimise data protection risk through detailed network segmentation, ensuring scalable configuration and consistent protection across the infrastructure.

Application-level protection:

- Amazon Shield is a managed AWS Cloud service for DDoS (Distributed Denial of Service) protection against all known infrastructure (Layer 3 and 4) attacks.
- Amazon Web Application Firewall (WAF) protects the web apps by filtering, monitoring and blocking any malicious HTTP/S traffic on layer 7. Protect against common web exploits that may affect availability, compromise security or consume excessive resources.

Network-level protection:

- AWS Network Firewall to create firewall rules that provide fine-grained control over network traffic and easily deploy firewall security across our VPCs
- Network ACLs (NACLs) - network access control list (ACL) allows or denies specific inbound or outbound traffic at the subnet level
- Amazon Route 53 - Resolver DNS Firewall is a feature that allows us to quickly deploy DNS protections across all our Amazon Virtual Private Clouds (VPCs)
- Encryption Protection refers to the use of encryption techniques to secure and safeguard sensitive information from unauthorized access or interception
- AWS Key Management Service (AWS KMS) create, manage, and control cryptographic keys. AWS KMS uses FIPS 140-2 Level 3
- AWS Secret Manager manages, retrieves, and rotates database credentials, API keys, and other secrets throughout their lifecycles.

All services are also protected by CrowdStrike services at different levels. All services in AWS use integrations between Crowdstrike Cloud Security and AWS services like Security Hub, Control Tower and Firewall Manager to monitor all the activity in our environments. Using Crowdstrike Insight XDR, CMI have visibility in case of attacks and other threats and notifying any alerts to Crowdstrike Falcon Overwatch (Security Operations Center - SOC) to immediately address potential threats on a 24/7/365 basis. CMI protect all assets using Falcon Intelligence, including CMI endpoints, to make sure all systems are protected and reduce to zero the option to be attacked. Additionally, using Falcon Data Protection as a DLP solution, CMI is protected against any kind of ransomware or other threat and reduce the possibility of any data leak.

Authentication

The combination of Auth0 and AWS offers real benefits for CMI’s application. With AWS, CMI has a powerful and scalable infrastructure to support the application workload. With Auth0, CMI has an identity architecture that scales with the application to meet their IAM needs. They have an easy way to federate their application with several customer identity providers using SAML or OIDC.

7.13 Monitoring

A trial steering committee (TSC) will be formed before the start of recruitment consisting of an independent chair, at least two independent members, and members of the research team (as non-voting members). A charter will be written and agreed upon by members outlining the remit and functioning of the TSC, including meeting frequency. The TSC will review recruitment data (and compare to pre-specified targets) and pooled information about participant characteristics and safety (adverse events), will not be presented with summaries split by arm.

To monitor safety during the trial, therefore, we will form an Independent Executive Committee. Before each TSC meeting, the executive committee will review numbers of adverse events and serious adverse events (see definitions below) by arm and make recommendations in writing to the TSC chair (e.g., if the committee feels the trial needs to be stopped). The TSC chair would then raise these issues for discussion and decision by the TSC. All reports (those of the TSC and executive committee) will be stored in the trial master file (TMF).

There are no interim analyses or audits of trial conduct planned.

7.14 Adverse events

7.14.1 Definitions

Adverse Event (AE)	Any untoward medical (either physical or mental) occurrence in a participant, including occurrences which are not necessarily caused by or related to this intervention.
Adverse Reaction (AR)	Any adverse event that is related to the allocated intervention. ARs in this study may be related to the intervention (push notifications, conduct of oral saliva tests, receipt of financial incentives) but not related to receiving OAT, as these are part of routine treatment.
Unexpected Adverse Reaction (UAR)	An adverse reaction the nature and severity of which is not consistent with the information known about the trial intervention in question.
Serious adverse Event (SAE), Serious Adverse Reaction (SAR) or Unexpected Serious Adverse Reaction (USAR)	Any adverse event, adverse reaction or unexpected adverse reaction, respectively, that:<br>• Results in death;<br>• Is life-threatening (with an immediate not hypothetical risk of death at the time of the event);<br>• Required hospitalisation or prolongation of existing hospitalisation (but not including elective hospitalisation for pre-existing condition);<br>• Results in a new persistent or new significant disability or incapacity defined as:<br>i. Severe: a significant deterioration in the participant's ability to carry out their important activities of daily living (e.g. employed person no longer able to work, caregiver no longer able to give care, ambulant participant becoming bed bound);<br>ii. Persistent: 4 weeks continuous duration;<br>• Any other important medical condition which, though not included in the above, may jeopardise the participant and

	may require medical, psychological or surgical intervention to prevent one of the outcomes listed;<br>• Any new episode of deliberate self-harm.

7.14.2 Adverse event reporting

We will monitor non-serious adverse events, serious adverse events, and serious adverse reactions to trial interventions, serious deterioration, and active withdrawals from treatment. Keyworkers will be asked to record (on CRF) and notify us if they are aware of any adverse events or active withdrawals from treatment. We will contact key workers once a week to monitor possible adverse reactions. These will be recorded in a specific SPSS database, stored on a secure KCL drive, and reported at each DMEC meeting.

Reporting responsibilities for AE/SAEs

Keyworkers and researchers will be asked to record any adverse events. Any Serious Adverse Events should be reported to the PI within 24 hours.

Any SAEs related to the intervention will be reported to the Research Ethics Committee within 15 days.

7.15 GDPR compliance

Data stored in the trial database will include participants unique PIN number, initials and date of birth. The trial database will only be accessible to the Principal Investigator (CAG). Data will be stored in password protected KCL network. At the point of giving consent, participants will be told how their personal data is going to be used, how it will be stored, and for how long. The trial data will be registered on the King's Data Protection Registration.

7.16 Data management

Researchers at King's College London (KCL) will collect data from the baseline and follow-up interviews on paper case report forms. These forms will be stored at KCL and entered into an SPSS database. Data from the app will also be exported into SPSS. KCL researchers and statisticians will develop the database and perform range checks during data entry. Accuracy will be verified by comparing 10% of entries to the paper case report forms. The SPSS databases will be stored on a secure drive at KCL and subject to version control. Only members of the KCL research team will have access to the data.

Research data will be archived in accordance with King's College London guidance. More specifically, digital files will be stored in SharePoint and then archived in King's Open Research Data System (KORDS). KORDS is a research data repository, providing long-term storage and access for datasets at the end of the study. Physical documents will be stored onsite in the Addictions Department at King's College London. They will then be archived with Corporate Records Management.

Each site will be responsible for its own archiving arrangements (as per their Trust/local policies).

7.17 End of study

The end of the study refers to the point at which trial databases are locked for analyses.

7.18 Dissemination, Outputs & Impact

We will provide a plain English summary of our findings to patients participating in the study. We will also provide summaries to drug services and via SLaM NHS Trust. We will publish our findings in open-access peer-reviewed journals.

7.19 Intellectual Property

The following IP arrangements are in place:

- Non-severable Foreground IP generated from CMI-owned Background IP and any improvements to Background IP (CMI app) will belong to CMI. All other Foreground IP and arising know-how is owned by King's College London.
- Research data (results and analysed data from participants) is owned or controlled by King's College London. Raw participant data is controlled by KCL or SLaM (where co-sponsored).
- In these studies, King's College London will grant to CMI a non-exclusive licence to use the Research data for non-commercial R&D purposes. A separate commercial licence will be required for CMI to use the research data for commercial purposes with appropriate revenue to KCL.
- King's College London and SLaM (if co-sponsored) will need to inform participants if CMI will receive raw data (anonymised data collected from participants under the studies). No identifiable personal data would be required to be shared with CMI.

7.20 Progression criteria

Progression to a larger confirmatory trial will be evaluated based on the progression criteria described below. However, not achieving these criteria does not necessarily indicate unfeasibility of a future trial but underlines changes that need to be made to recruitment procedures, attendance record keeping and resources for follow-up. These include:

	Green	Amber	Red
Number of eligible patients enrolled over the 6-month recruitment period	25, at least 12/per arm	≥15, at least 7/arm	<15
Percentage of screened patients who are eligible for inclusion in the feasibility trial.	≥50%;	≥30%;	<30%
Adherence to the intervention based on percentage of requested uploads returned	≥50% in CM arm	≥30% in CM arm	<30% in CM arm

Percentage of urine drug screenings completed, overall	≥70% in all arms	≥50% in all arms	<50% in all arms
Number and percentage attending follow-up interview at end of 12-week intervention period (compared to number randomised)	≥70%	≥50%	<50%

8 Workstream 3: Experience Sampling Methodology (ESM)

8.1 Aims and objectives

The aim of this workstream is to explore the dynamic interplay of psychological, environmental, contextual factors and the mCM intervention on influencing heroin use in real-time. This specific aim is to answer the following research question: What impact does CM and related psychological, environmental, and contextual factors have on heroin use behaviour?

Identifying potential mechanisms and how they impact clinical outcomes can help better understand why individuals continue to use heroin when in opioid agonist treatment and how CM interventions could be leveraged to optimise outcomes.

To address this research question, the following objectives are:

Objectives:

1. Identify key constructs and variables influencing heroin use, including psychological, environmental, and contextual factors.
2. Select an ESM format (text message, app-based) based on the preferences of the target group to maximize engagement and compliance.
3. Determine the optimal sampling strategy (event-contingent or time-contingent) to capture real-time experiences of participants.
4. Develop questions that capture the dynamic interplay between psychological, environmental, and contextual factors and the mCM intervention on influencing heroin use in real-time.
5. Consider response formats (e.g., Likert scales, multiple-choice options, open-text responses) to ensure ease of comprehension and accurate reporting.
6. Ensure that questions and prompts are relevant to participants' everyday experiences and contexts, capturing the nuances of their interactions with the mCM intervention and heroin use.
7. Develop a comprehensive understanding of the mCM intervention's impact on heroin use behaviours and associated psychological, environmental, and contextual factors.

8.2 Methodology

8.2.1 Design

ESM will be used to monitor participant’s thoughts, feelings, behaviours, and interactions with the mCM intervention. ESM is a specialist diary-based questionnaire used to gather momentary data from participants during their everyday life (22). In contrast to traditional

questionnaires and clinical interviews delivered at the start and end of the study period, ESM is a self-reported assessment that is completed during participant's everyday life, continuously capturing symptoms and thoughts in real time. ESM will be valuable for understanding the experiences of participants receiving the mCM intervention. Questionnaires will take approximately 5 minutes to complete.

8.2.2 Participants

Following the completion of trial outcome assessments (12-week follow-up), participants assigned to the mCM arm will be invited to receive the mCM intervention for an additional two-week period, during which time they will complete daily ESM questionnaires. Participants will be reimbursed for responding to questionnaires. The amount earned will depend on response rates (£50 for 100%; £40 for >85%; £30 for > 70%, £20 for >55%, £10 for >40%).

8.2.3 Data collection

The data collection process for the Experience Sampling Methodology (ESM) involves participants in receipt of mCM arm completing daily questionnaires designed to capture momentary data on their thoughts, feelings, behaviours, and interactions with the mCM intervention and heroin use. See Appendix B for example questions. Participants will be prompted to respond to questions in real-time during their everyday lives, using either a text message or app-based format. A mixed design approach will be adopted, allowing for both event-contingent (participant-initiated) and time-contingent (system-initiated) sampling strategies. This enables the recording of events as they occur, such as interactions with the mCM intervention or instances of heroin use, while also gathering data pre- and post-event to provide context and capture missed events.

8.2.4 Data analysis

ESM data will be analysed using mixed effect (i.e., multilevel) models to account for the hierarchical structure of the data (i.e., repeated observations nested within individuals). Multilevel models allow for multiple levels of data to be considered without the need for aggregation and can be used to examine variation at each level of nesting. They can accommodate the nested structure of ESM data, are valid for unbalanced data sets and can be extended to fit complex covariance structures arising in the data.

9 Project timeline

The study is being undertaken over a 38-month period. See Table 1 for a detailed timeline.

Table 1. Gantt Chart

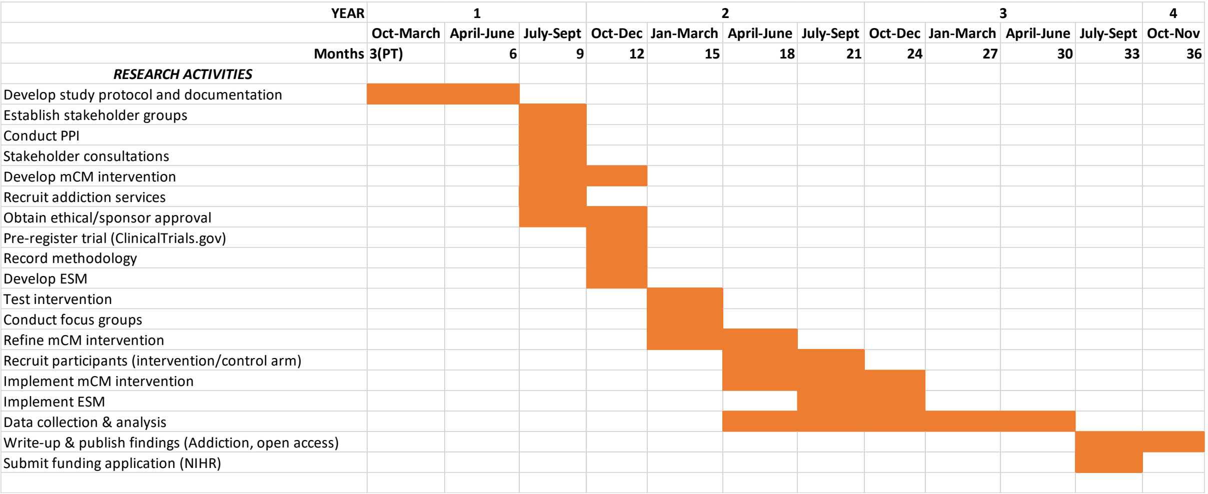

10 Project management

CAG will manage the project including the day-to-day running of the study. A project management team consisting of CAG, NM, EC and JS meet monthly. A Trial Steering Committee will be convened, and an executive committee will review safety information by arm before each TSC (see details above). A PPI advisory group will meet three times during the study: prior to app development, before the start of recruitment and at the end of data collection to help interpret the findings.

11 Ethics/Regulatory Approvals

As in any research involving participants, we will have arrangements in place for obtaining informed consent. We expect the drug service staff who are already known to patients will provide them with clear information about the study and take informed written consent. Taking part in the research is optional and will not affect a service user’s usual treatment. This will be made clear to participants. Our previous work found no/low risks from mCM (23). Participants are seen as usual by drug services/receive usual treatment. Therefore, we believe risks are low, and we will monitor safety outcomes during the trial.

HRA and NHS REC ethical approval will be sought before the study commences.

12 Protocol amendments

Protocol modifications will be discussed and approved by the TSC. All amendments will be reviewed and approved by the R&D office for SLaM/IoPPN (sponsor).

13 References

1. European Monitoring Centre for Drugs and Drug Addiction. EMCDDA Statistical bulletin 2019. 2019.

2. Public Health England. Adult substance misuse treatment statistics 2020 to 2021: report. In: Statistics N, editor. 2021.

3. Lussier JP, Heil, S. H., Mongeon, J. A., Badger, G. J., & Higgins, S. T. A meta-analysis of voucher-based reinforcement therapy for substance use disorders. Addiction. 2006;101(2), 192-203.

4. Griffith JD, Rowan-Szal GA, Roark RR, Simpson DD. Contingency management in outpatient methadone treatment: a meta-analysis. Drug & Alcohol Dependence. 2000;58(1):55-66.

5. Ainscough TS, McNeill A, Strang J, Calder R, Brose LS. Contingency Management interventions for non-prescribed drug use during treatment for opiate addiction: A systematic review and meta-analysis. Drug & Alcohol Dependence. 2017;178:318-39.

6. Gates PJ, Sabioni, P., Copeland, J., Le Foll, B., & Gowing, L. Psychosocial interventions for cannabis use disorder. Cochrane Database of Systematic Reviews. 2016;5.

7. Benishek LA, Dugosh, K. L., Kirby, K. C., Matejkowski, J., Clements, N. T., Seymour, B. L., & Festinger, D. S. Prize-based contingency management for the treatment of substance abusers: A meta-analysis. Addiction. 2014;109(9):1426-36.

8. Petry NM, Rash CJ, Byrne S, Ashraf S, White WB. Financial reinforcers for improving medication adherence: findings from a meta-analysis. The American journal of medicine. 2012;125(9):888-96.

9. Bolívar HA, Klemperer EM, Coleman SR, DeSarno M, Skelly JM, Higgins ST. Contingency management for patients receiving medication for opioid use disorder: a systematic review and meta-analysis. JAMA psychiatry. 2021;78(10):1092-102.

10. National Institute for Clinical Excellence. Drug misuse in over 16s: psychosocial interventions. In: Excellence MNIfHaC, editor. 2007.

11. Marsch LA, Dallery J. Advances in the Psychosocial Treatment of Addiction. The Role of Technology in the Delivery of Evidence-based Psychosocial Treatment. Psychiatric Clinics of North America. 2012;35(2):481-93.

12. Dallery J, Raiff BR. Contingency management in the 21st century: technological innovations to promote smoking cessation. Substance Use & Misuse. 2011;46(1):10-22.

13. Getty CA, Morande A, Lynskey M, Weaver T, Metrebian N. Mobile telephone-delivered contingency management interventions promoting behaviour change in individuals with substance use disorders: a meta-analysis. Addiction. 2019;114(11):1915-25.

14. Getty CA, Weaver, T., & Metrebian, N. A qualitative exploration of patients' experience of mobile telephone-delivered Contingency Management (mCM) to promote adherence to supervised methadone. Drug & Alcohol Review. 2022;Accepted/In Print.

15. Getty CA, Weaver T, Lynskey M, Kirby KC, Dallery J, Metrebian N. Patients' beliefs towards contingency management: Target behaviours, incentives and the remote application of these interventions. Drug and Alcohol Review. 2021.

16. Michie S, Van Stralen MM, West R. The behaviour change wheel: a new method for characterising and designing behaviour change interventions. Implementation science. 2011;6:1-12.

17. Bradbury K, Watts S, Arden-Close E, Yardley L, Lewith G. Developing digital interventions: a methodological guide. Evidence-Based Complementary and Alternative Medicine. 2014;2014.

18. Brooke J. SUS-A quick and dirty usability scale. Usability evaluation in industry. 1996;189(194):4-7.

19. Darke S HW, Wodak A, Heather N, Ward J. Development and validation of a multidimensional instrument for assessing outcome of treatment among opiate users: the Opiate Treatment Index Addiction. 1992;87:733–74217.

20. Zigmond AS SP. The hospital anxiety and depression scale. Acta Psychiatr Scand. 1983;67:361–70.

21. Miller WR, Tonigan JS. Assessing drinkers' motivation for change: the Stages of Change Readiness and Treatment Eagerness Scale (SOCRATES): American Psychological Association; 1997.

22. Csikszentmihalyi M, Larson R. Validity and reliability of the experience-sampling method. The Journal of nervous and mental disease. 1987;175(9):526-36.

23. Metrebian N, Weaver, T., Pilling, S., Goldsmith, K., Carr, E., Shearer, J., Woolston-Thomas, K., Tas, B., Getty, C.A., Cooper, C. and van der Waal, R. Mobile telephone delivered contingency management for encouraging adherence to supervised methadone consumption: feasibility study for an RCT of clinical and cost-effectiveness (TIES). Pilot and Feasibility Studies. 2021;7.
